# Interaction of *SARS-CoV-2* spike protein with angiotensin converting enzyme inhibitors and selected compounds from the chemical entities of biological interest

**DOI:** 10.1186/s43088-021-00138-3

**Published:** 2021-08-25

**Authors:** Suleiman Aminu, Mohammed Auwal Ibrahim, Abdullahi Balarabe Sallau

**Affiliations:** Department of Biochemistry, Ahmadu Bello University, Zaria, Nigeria

**Keywords:** *SARS-CoV-2*, Spike protein, Angiotensin converting enzyme, Flavonoids

## Abstract

**Background:**

Recent COVID-19 outbreak has prompted the search of novel therapeutic agents to treat the disease. The initial step of the infection involves the binding of the virus through the viral spike protein with the host angiotensin converting enzyme 2 (ACE2). In this study, the interaction of some ACE or ACE2 inhibitors and their analogues as well as selected compounds with the viral spike protein as a strategy to hinder viral-ACE2 interaction were investigated. *SARS-CoV-2 *spike protein as well as the ligands were retrieved from protein databank and ChEBI database respectively. The molecules were prepared before initiating the virtual screening using PyRx software. Discovery studio was used to further visualize the binding interactions between the compounds and the protein.

**Results:**

The ACE inhibitors and their analogues fosinopril (1-), fosinopril and moexipril have the best binding affinity to the protein with binding energies < − 7.0 kcal/mol while non-flavonoid stilben-4-ol binds with free binding energy of − 7.1 kcal/mol. Others compounds which belong to either the flavonoids, terpenes and alkaloid classes also have binding energies  < − 7.0 kcal/mol. Such high binding energies were enhanced via hydrogen bond (h-bond) interactions in addition to other interactions observed between the compounds and the amino acid residues of the protein.

**Conclusions:**

The ACE inhibitors and their analogues as well as the selected compounds could serve as inhibitors of the spike protein as well as lead in drug discovery processes to target the *SARS-CoV-2 *virus.

## Background

Coronaviruses (CoV) are large family of zoonotic viruses known to cause illnesses ranging from a common cold to more severe conditions such as respiratory syndrome [1]. The recent outbreak of a novel CoV virus [*SARS-CoV-2*] disease which originated from Wuhan, China and progressively spread to all parts of the world prompted the World Health Organization (WHO) to declare the disease as a pandemic, named COVID-19 [2]. The virus affects the respiratory system and causes breathing difficulties with chronic pneumonia, severe respiratory syndromes in addition to fever and kidney failure which may lead to death of the patients [3]. Currently, there are no specific known drugs against the disease and the global attention focused on the scientific community for a possible solution.

The observed symptoms of COVID-19 occur as a result of the binding interaction between the virus spike protein and the host Angiotensin Converting Enzyme 2 (ACE2) receptors [4] located on the alveolar cells surfaces in the lungs [5]. This process facilitated the entry of the virus into the infected host cell and therefore, blocking the *SARS-CoV-2* S protein could ultimately prevent the viral-ACE2 interaction and renders the virus non-infectious. Interestingly, the crystal structure of the spike protein has been elucidated and released [6]. The trimeric protein contains S1 and S2 subunits in addition to receptor binding domain that altogether play a role in the binding interaction. Such a fascinating scientific effort is an important milestone in the search for drugs or vaccines against the disease [7]. This is because the spike protein has been considered to be the most appealing drug and/or vaccine target by scientists. Moreover, the current search for COVID-19 chemotherapeutics relied on drug repurposing and/or repositioning [8]. In these regards, a number of databases are available for such purposes and among them, the Chemical Entities of Biological Interest (ChEBI) is one of the most versatile [9]. It consists of freely available molecular entities focused on small chemical compounds as part of Open Biomedical Ontologies effort which is a resource of the US National Center for Biomedical Ontology.

As earlier noted, the important role of the ACE2 in mediating the viral entry into the host cells could suggest that ACE or ACE 2 inhibitors and their analogues in addition to similar compounds from the ChEBI database could be explored for drug repurposing research against the viral spike protein. Additionally, scientific investigations have showed natural products, especially flavonoid and non-flavonoid phenolics, terpenes and alkaloids as promising therapeutic candidates against the *SARS-CoV-2 *which might be explored as possible inhibitors of the viral spike protein [10, 11]. In this study, we investigated the binding and interaction of ACE or ACE2 inhibitors and their analogues as well as, flavonoids, non-flavonoid phenolics, terpenes and alkaloids available in ChEBI with the *SARS-CoV-2 *spike protein using molecular docking. The result obtained could add to the wealth of information for the ongoing search of lead compounds against COVID-19 by the scientific community.

## Methods

### *Retrieval and preparation of* SARS-CoV-2*spike protein*

The Cryo-EM structure of the trimetric *SARS-CoV-2 *spike protein containing *N*-acetyl-*D*-glucosamine (PDB ID: 6vsb) was extracted from RCSB Protein Data Bank (http://www.rcsb.org). The protein was prepared for molecular docking using Chimera docking software version 1.14 (https://www.cgl.ucsf.edu/chimera/download.html). In the Chimera, chains B and C in addition to the NAG contained in the protein structure were removed. Subsequently, the remaining chain A was docked prep by adding h-bonds while all other settings were set as default. After preparation, the chain A of *SARS-CoV-2 *spike protein was saved in a PDB format and transferred to PyRx virtual screening software (https://pyrx.sourceforge.io/). Therein, the molecule was prepared as autodock molecule and stored in pdbqt format.

### Retrieval and preparation of ligands 3-D structures

All the ligands used for the docking experiment were retrieved from ChEBI database. Additionally, the 3D structures of twenty seven (27) ACE inhibitors, 2 ACE2 inhibitors and their structural analogues were retrieved from the database. Moreover, nineteen (19) non-flavonoid phenolics, twenty three (23) flavonoids, twelve (12) terpenes and ten (10) alkaloids were also retrieved from the database for subsequent docking experiment. The compounds were retrieved as SDF files and were imported to the PyRx virtual screening software (https://pyrx.sourceforge.io/). Following retrieval, ligands were prepared by applying universal force field (UFF) to minimize all the minimum energy for each configuration. Thereafter, the ligands were converted to the pdbqt format (autodock ligands) in preparation for docking.

### Molecular docking

For the molecular docking experiment, the autodock ligands were docked against the *SARS-CoV-2 *spike protein. This was initiated by commanding the Vina wizard to commence the docking process followed by maximizing (in the absence of ligand) the Auto Grid boxes center (*x*, *y*, *z* coordinates 206.048, 223.411, 226.7943) and dimension (*x*, *y*, *z* coordinates 82.8917, 79.9188, 168.0678) to cover the entire protein and accommodate ligand to move freely and select the best binding site. In the PyRx software, the binding energies of the interactions which indicate the best predicted binding modes to the proteins [12, 13] were computed and retrieved in Microsoft excel.

### Structural analysis and visualization

To further visualize the docking result and deduce the possible receptor- ligand interaction, Discovery studio visualizer (https://www.3dsbiovia.com/products/collaborative-science/biovia-discoverystudio/visualization-download.php) was used for the obtained docking results contained in the working directorate. The 2-D interactions were visualized in order to determine possible bond interactions between amino acid residues of the *SARS-CoV-2 *spike protein and the ligands. Bond lengths were also calculated using the software.

## Results

Docking of ACE-2 inhibitors and analogues against the *SARS-CoV-2 *spike protein showed fosinopril (1-), fosinopril, moexipril and novacine to have the best affinity to the protein with free binding energies > − 7.0 kcal/mol (Table 1) while stilben-4-ol had the best binding affinity among the phenolics with a free binding energy of -7.1 kcal/mol. The flavonoids, cudraflavone, artocarpine, papyriflavonol, 7-hydroxyflavone, primetin, scutellerein, quercetagetin, robinetin, violanthin, isoharmetin-3-O-rutinoside, cirsilineol, tectochrysin, frutinone A, pelargonodine-3-O-rutinoside betaineand narirutin had binding energies ≥ − 7.0 kcal/mol respectively. Moreover, among the terpenes and alkaloids, rediocide A, phytolaccoside B, mesulgine and hapalindole have highest binding affinity to the protein than others (Table 1). Among the selected classes of compounds, it was noted generally that the number of flavonoids with good binding affinity to the *SARS-CoV-2 *spike protein was the highest (Table 1).

**Table 1:** Binding energies (B/E) of compounds against *SARS-CoV-2* spike protein

ACE/ACE 2 inhibitors/derivatives	B/E (Kcal/mol)	Non-flavonoid Phenolics	B/E (Kcal/mol)	Flavonoids	B/E (Kcal/mol)	Terpenes	B/E (Kcal/mol)	Alkaloids	B/E (Kcal/mol)
Captopril	− 5.3	2-methoxy-6-(all trans-nonaprenyl)phenol	− 5.5	Quercetin	− 6.7	**Rediocide A**	− **8.0**	**Mesulergine**	− **7.5**
Lividomycin A	− 6.6								
Lividomycin B	− 6.9								
Captopril disulphide	− 5.7	2-polyprenylphenol	− 6.1	**Cudraflavone**	− **7**	α-pinene	− 5.1	Daphane	− 6.2
3-acetylthioisobutyric acid	− 4.5	3,5-dimethyl-4-(methylSulfanylphenol)	− 5.2	**Artocarpin**	− **7.2**	β-pinene	− 6.1	3-pyridylacetic acid	− 5.6
Benazepril	− 5.3	4-methylaminophenol	− 5.1	**Papyriflavonol**	− **7.2**	Nerol	− 4.1	Precondylocarpine acetate	− 6.9
Benazepril (1 +)	− 6.8	Thymol	− 5.9	**7-hydroxyflavone**	− **7.2**	Farnesol	− 5.4	Dihydro precondylocarpine acetate	− 6.3
Zofenoprilat	− 5.8								
Benazepril hydrochloride	− 6.1	Propofol	− 5.5	Galangin	− 6.8	Phytol	− 5.8	5-aminopentanal	− 3.4
Benazeprilat	− 6.8	**stilben-4-ol**	− **7.1**	**Primetin**	− **7.3**	**Phytolaccoside B**	− **7.1**	Dehydrosecodine	− 5.8
Enalapril	− 5.7	Triclosan	− 5.6	**Scutellarein**	− **7.6**	α-ionone	− 6.0	Secodine	− 6.1
Enalapril malate	− 4.7	Aspergillusene	− 5.2	Tangeratin	− 5.8	β-ionone	− 6.1	Lupanine	− 6.2
Enalaprilate (anhydrous)	− 6.7	bisphenol F diglycidylether	− 5.7	Cirsiliol	− 6.5	Dehydrovomifoliol	− 5.2	**Hapalindole**	− **7.8**
Enalaprilat dehydrate	− 1.9	2-acetylphenol	− 6	**Cirsilineol**	− **7.5**	Linalool	− 4.2		
Fosinopril	− **7.1**	2-ethoxyphenol	− 4.7	Nevadensin	− 6.1	Geranylacetate	− 4.4		
Fosinopril (1-)	− **7.2**	mycophenolic acid	− 6.8	**Quercetagetin**	− **7**				
Fosinoprilat	− 5.6	Neotriptophenolide	− 6.6	**Robinetin**	− **7.7**				
Lisinopril	− 5.4	2,3,4,5-tetrachlorophenol	− 5.5	Sinensetin	− 6.2				
Lisinopril dehydrate	− 1.9	2-acetamidophenolsulfate (1-)	− 5.1	**Tectochrysin**	− **7.5**				
				**Frutinone A**	− **7.1**				
Moexipril	− **7**	2-acetamidophenolsulfate	− 5.3	**Violanthin**	− **7.7**				
Methyl-1-methyl-5-oxo-prolinate	− 4.7			**Narirutin**	− **8.0**				
				**Pelargonidine 3-O-rutinoside betaine**	− **7.6**				
Moexipril hydrochloride	− 6.2	Triptophenolide methylether	− 6.5	Wogonin	− 6.9				
Quinapril	− 6.9	2,3,6-trichloro-4-hydroxyphenolate	− 5.2	Meta-hydroxylphenylhydracrylic acid	− 5.4				
Quinapril hydrochloride	− 5.8								
	− 5.8								
Quinapril (1 +)	− 6.2			**Isoharmnetin-3-o-rutinoside**	− **7.1**				
Quinaprilat	− 6.8								
Cys-Pro	− 4.4								
Novacine	− **7.3**								

Values indicated in bold have binding energy < − 7.0 kcal/mol

Visualization of the receptor-ligand interaction revealed the presence of h-bond interactions which contributed to the observed binding energies realized in the docking (Table 2). Among the ACE inhibitors and their analogues, fosinopril (1-) have two h-bond interactions with His1058 and Gln853 residues of the protein while moexipril and novacine formed a single h-bond with His1058 and Ser591 residues respectively. In the case of fosinopril and the non-flavonoid phenolics stilbene-4-ol, there were absence of h-bond interaction with the protein. Similarly, all the flavonoids formed 1 or more h-bond interactions. Among the flavonoids, quercetagetin formed 5 h-bonds with Phe342, Asn343, Ser373, Ser375 and Arg509 residues of the protein followed by scutellerein and isoharmetin-3-O-rutinoside with 4 h-bonds each. Nonetheless, the terpenes, rediocide A and phytolaccoside B formed 2 h-bonds interactions with the protein mainly involving Ala713, Tyr1047, Gln564 and Phe565 respectively while the alkaloid, hapalindole formed 1 h-bond interaction with Met731 (Table 2).

**Table 2:** Hydrogen (H_2_) bonds and interacting amino acid residues between the compounds and *SARS-CoV-2* spike protein

Compounds	Number of H_2_ bonds	Bond length (A^0^)	Interacting AA residues
Fosinopril	0		
Fosinopril (1-)	2	2.76, 2.41	His1058, Gln853
Moexipril	1	2.57	His1058
Novacine	1	1.91	Ser591
Stilben-4-ol	0		
Cudraflavone	1	2.13	Val608
Artocarpin	2	1.81, 2.31	Arg1107
Papyriflavonol	3	2.32, 2.39, 2.50	Asp796, Ile896, Phe898
7-hydroxyflavone	1	2.9	Tyr160
Primetin	1	1.93	Thr778
Scutellarein	4	2.11, 2.19, 2.25, 2.76	Lys733, Gln774, Thr778
Quercetagetin	5	2.36, 2.35, 2.84, 2.61, 2.67	Phe342, Asn343, Ser373, Ser375, Arg509
Robinetin	1	2.41	Ala123
Violanthin	2	2.15, 2.21	Ser591, Phe592
Isoharmnetin-3-o-rutinoside	4	1.93, 2.15, 2.41, 2.57	Phe59, Thr29, Ser60
Cirsilineol	2	2.28, 2.65	Ser730, Pro1057
Tectochrysin	2	2.17, 2.46	Thr778, Lys733
Frutinone A	2	2.52, 3.03	Leu223, Ile285
Narirutin	2	2.10, 2.77	Leu828, Ala956
Pelargonidine-3-O-rutinoside betaine	2	1.91, 2.03	Phe823, Asn824
Rediocide A	2	2.42, 2.88	Ala713, Tyr1047
Phytolaccoside B	2	2.29, 2.52	Gln564, Phe565
Mesulergine	0		
Hapalindole	1	2.85	Met731

**Fig. 1:**
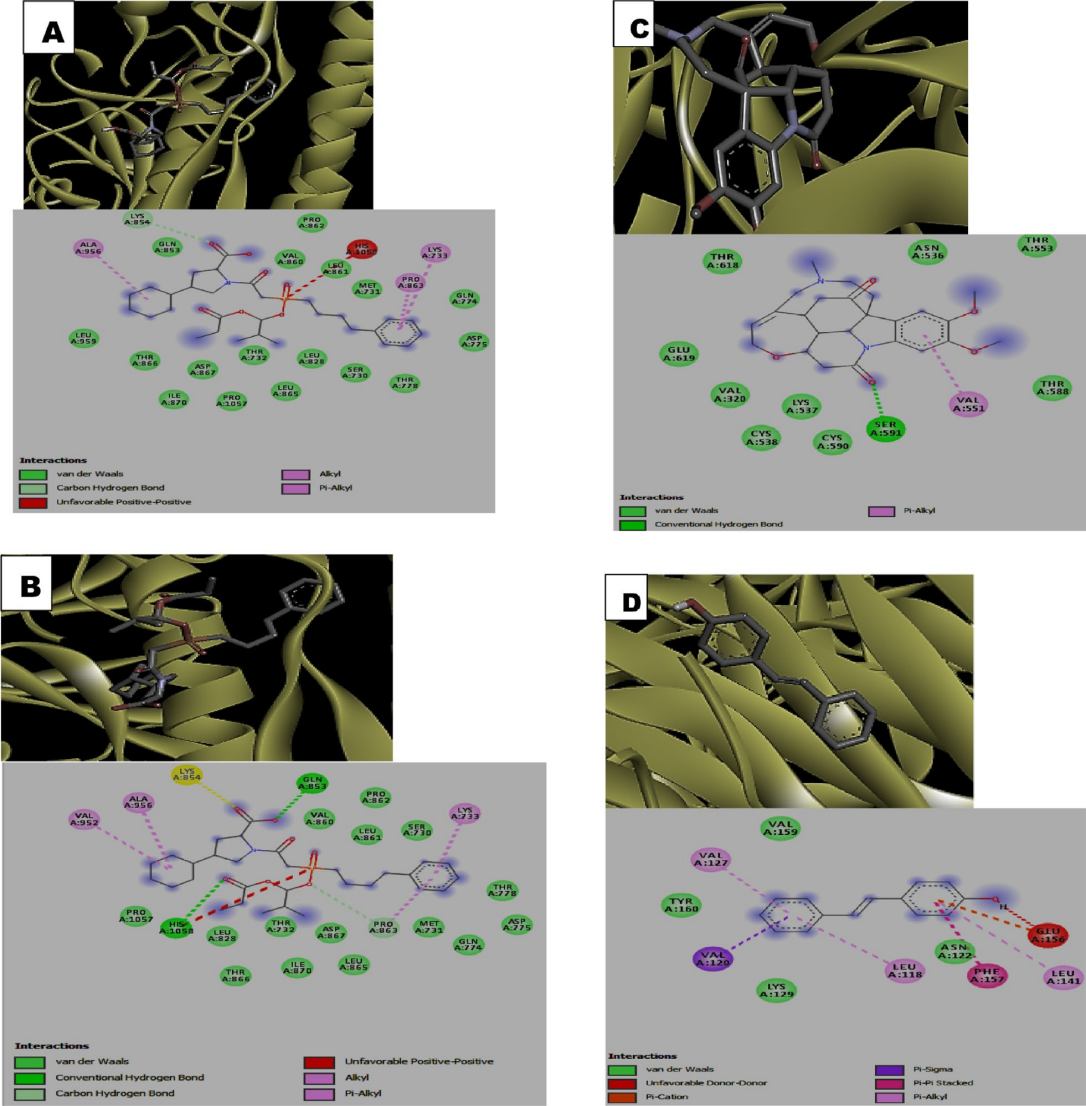

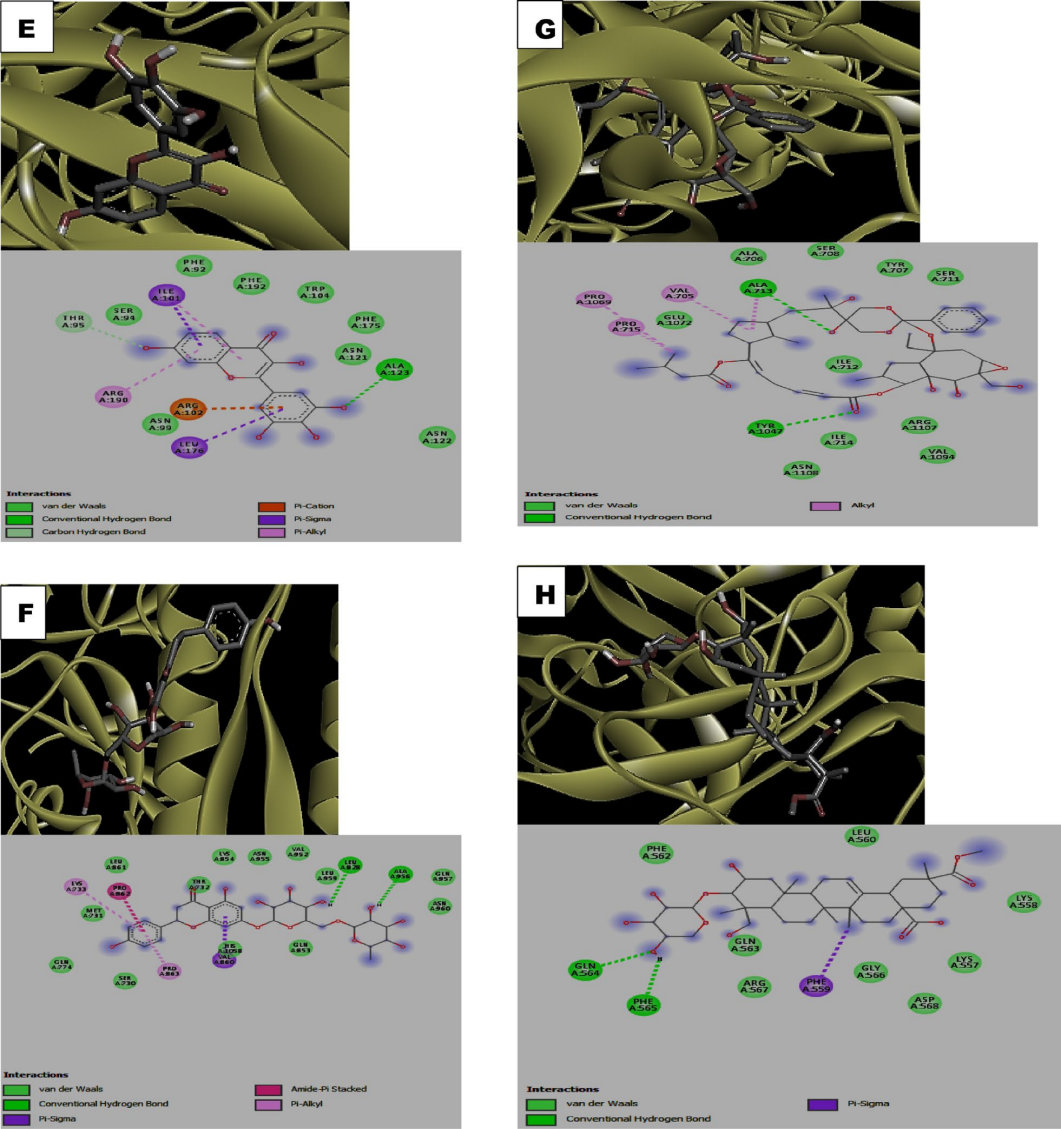

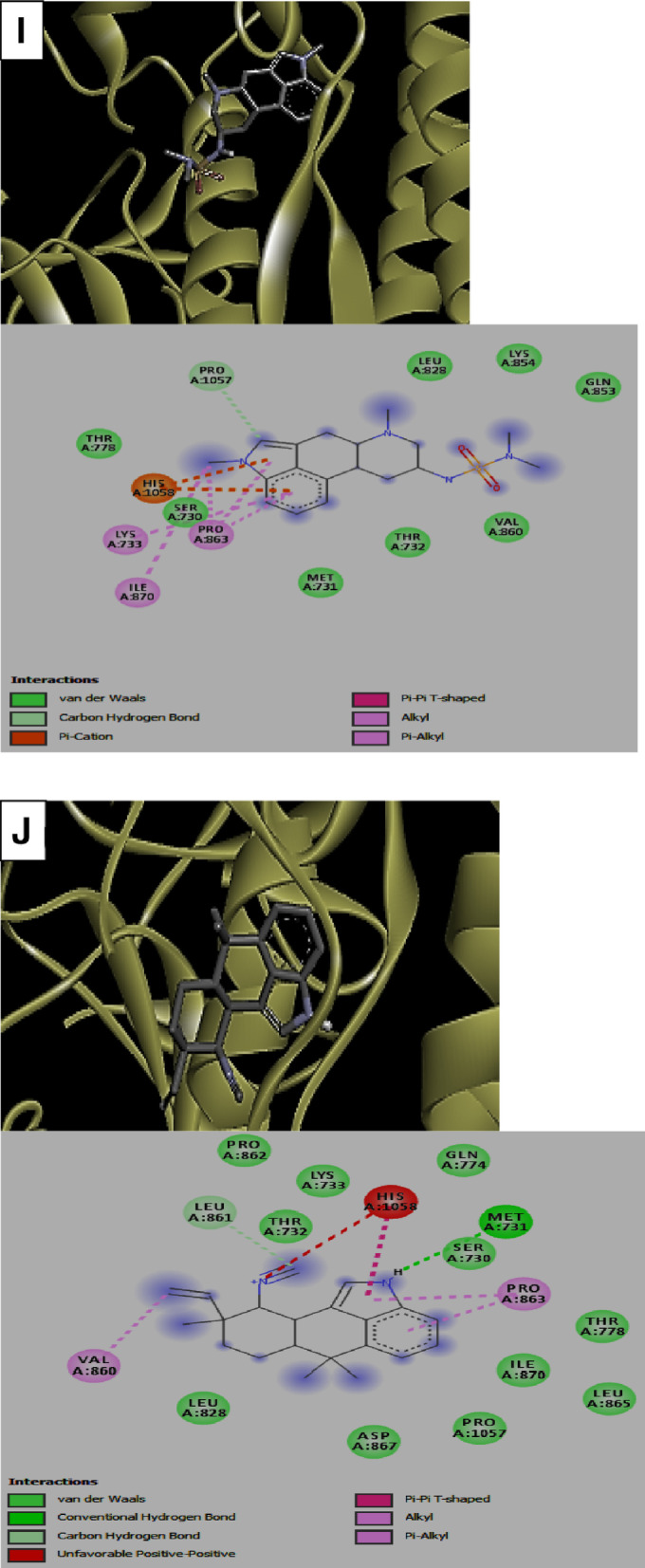
3-D structure representation of **A** Fosinopril **B** Fosinopril (1-) **C** Novacine **D** Stilbene-4-ol **E** Robinetin **F** Narirutin **G** Rediocide A **H** Phytolaccoside B **I** Mesulergine **J** Hapalindole complexed with *SARS-CoV-2* spike protein respectively

In addition to the h_-_bond, other interactions such as van der Waal interactions, pi-Alkyl interactions, carbon-hydrogen interactions, pi- Sigma interactions among others were found to be critical to the high binding affinities of the compounds to the *SARS-CoV-2 *spike protein (Fig. 1). Noticeably, the ACE inhibitors (fosinopriland fosinopril (1-)) as well as the alkaloid hapalindole formed unfavorable positive-positive interaction with the protein while unfavorable donor-donor interaction was observed with the non-flavonoid stilbene-4-Ol (Fig. 1).

## Discussion

The increasing number of COVID-19 cases worldwide has become a public health concern that demand an urgent scientific attention [14, 15]. One of the important strategies to prevent the virus from exerting damage to the host is by blocking viral-host interaction which is mediated through the spike protein [16]. In this regard, PyRx software which is a very versatile and powerful tool was used in virtual screening of compounds from ChEBI database against *SARS-CoV-2* spike protein without initial validation. This is because the *SARS-CoV-2* spike protein was obtained through cryo-EM without any ligand and similar software was used in screening of antiviral compounds as potential inhibitors of *SARS-CoV-2* methyltransferase [17]. Although there were a lot of conflicting reports on the use of ACE2 inhibitors on COVID-19 patients [18, 19], our docking results showed fascinating binding interaction between the ACE inhibitors and the spike protein. The presence of phosphinate group in fosinopril and fosinopril (1-) suggests the reason for their observed high binding affinity than the other ACE inhibitors. The group has been reported to specifically bind to ACE and inhibit the production of angiotensin II [20]. The presence of h-bond interaction of fosinopril (1-) with the *SARS-CoV-2 *spike protein might have further contributed to its high binding affinity. Such interaction occurs at the S2 subunit of the protein since the His1058 and Gln853 residues are found within the region [21]. Similarly, moexipril and novacine formed h-bond interactions at the S2 and S1 subunit of the protein respectively. As the S1 and S2 subunits of the protein enhance receptor binding and viral fusion [22, 23], our result showed the possibility of the inhibitors to be used against the protein [24]. Meanwhile, it is worthy to state that two proline residues were added at the C-terminal fusion machinery of the *SARS-CoV-2* spike protein (6VSB) but that was not envisaged to affect the biochemical features of the protein because it was mainly useful during Cryo-EM process.

Natural compounds such as non-flavonoid and flavonoid phenolics, terpenes and alkaloids have gained a lot of attentions as they serve as lead during drug discovery processes [25]. Some of these compounds have been recently exploited as possible inhibitors against *SARS-CoV-2 *proteins [10, 26]. For the first time, investigation of such compounds against the viral spike protein might further support their efficacy against the virus.

The anti-viral effects of non-flavonoids and flavonoid phenolics have been reported in numerous studies [10, 27]. Most of their anti-viral effects have been known to occur via several mechanisms including the blockage of viral entry which is consistent with our findings [28]. High binding affinities in addition to observed h-bond interaction between the compounds showed their possible efficacy against the *SARS-CoV-2 *spike protein. Interestingly, most of the compounds interacted with the protein at the S2 sub-unit with the exception of robinetin and violanthin that interacted with the protein’s amino acid residues at S1 sub-unit. Specifically, the reported anti-viral effects of several non-flavonoids and flavonoid phenolics such as quercetin, artocarpin, tectochrysin and stilbene-4-Ol among others, against different human and animal viruses [29, 30] further support our investigations.

Terpenes and alkaloids are another class of compounds reported to possess anti-viral activities. For instance, β-pinene was found to masks herpes virus structure which is necessary for the viral entry into the host [31]. Such mechanism could explain the observed high binding interaction of terpenes with the *SARS-CoV-2 *spike protein. The presence of benzoic acid in the diterpenoid rediocide A and the triterpenoid nature of phytolaccoside B could suggest the higher binding interactions with the spike protein. Although there is a scanty information on the antiviral effects of the above mentioned compounds, many reports have proved the anti-influenza activities of benzoic acid derivatives [32] while triterpenoids have been known to exhibit a wide range of effects on respiratory viral infections [33, 34]. The ergoline in addition to sulfuric and oxo-acids structure of mesulergine as well as the indole ring of hapalindole alkaloids give them ability to interact reasonably well with the *SARS-CoV-2 *spike protein. Most of these compounds interacted with the S2 sub-unit of the protein.

## Conclusions

Based on our findings, it become paramount that ACE inhibitors and the selected compounds from the ChEBI database could interact reasonably well with the *SARS-CoV-2 *spike protein. Our data suggest that the compounds could be exploited as anti-COVID-19. Our future work will focus on the experimental validation of some of the findings in order to confirm the conclusions.

## Data Availability

Not applicable.

## Abbreviations

ACE2: Angiotensin converting enzyme 2
ChEBI: Chemical entities of biological interest
COVID-19: Novel coronovirus disease for 2019
NAG: *N*-Acetyl glucosamine
PDB: Protein database
